# Superclone Expansion, Long-Distance Clonal Dispersal and Local Genetic Structuring in the Coral *Pocillopora damicornis* Type *β* in Reunion Island, South Western Indian Ocean

**DOI:** 10.1371/journal.pone.0169692

**Published:** 2017-01-09

**Authors:** Pauline Gélin, Cécile Fauvelot, Vincent Mehn, Sophie Bureau, Héloïse Rouzé, Hélène Magalon

**Affiliations:** 1 UMR ENTROPIE (IRD, Université de La Réunion, CNRS), Laboratoire d’excellence-CORAIL, Université de La Réunion, St Denis, La Réunion; 2 UMR ENTROPIE (IRD, Université de La Réunion, CNRS), Laboratoire d’excellence-CORAIL, centre IRD de Nouméa, New Caledonia; National Cheng Kung University, TAIWAN

## Abstract

The scleractinian coral *Pocillopora damicornis* type *β* is known to present a mixed reproduction mode: through sexual reproduction, new genotypes are created, while asexual reproduction insures their propagation. In order to investigate the relative proportion of each reproduction mode in *P*. *damicornis* type *β* populations from Reunion Island, Indian Ocean, clonal propagation along the west coast was assessed through four sampling sites with increasing geographical distance between sites. Coral colonies were sampled either exhaustively, randomly or haphazardly within each site, and genotypic diversity was assessed using 13 microsatellite loci over a total of 510 *P*. *damicornis* type *β* determined *a posteriori* from their mtDNA haplotype (a 840 bp sequenced fragment of the Open Reading Frame). Overall, 47% of all the sampled colonies presented the same multi-locus genotype (MLG), a superclone, suggesting that asexual propagation is extremely important in Reunion Island. Within each site, numerous MLGs were shared by several colonies, suggesting local clonal propagation through fragmentation. Moreover, some of these MLGs were found to be shared among several sites located 40 km apart. While asexual reproduction by fragmentation seems unlikely over long distances, our results suggest a production of parthenogenetic larvae. Despite shared MLGs, two differentiated clusters were enclosed among populations of the west coast of Reunion Island, revealing the necessity to set up appropriate managing strategies at a local scale.

## Introduction

Asexual reproduction allows high population growth rates, successful genotypes propagation and energy savings due to the lack of mate search (*e*.*g*., [1]). However, contrary to sexual reproduction [2] which is more costly [3], asexual reproduction does not offer the opportunity to (1) produce recombinant types that might be advantageous [4, 5], (2) flush out deleterious mutations [6, 7], and (3) keep up with environmental modifications by increasing genetic diversity in populations [8]. Nevertheless, even with efficient DNA repair mechanisms (*e*.*g*., in bdelloid rotifers; [9]), most of asexual lineages need some occasional sexual recombination to counter the disadvantage of not reproducing sexually in order to persist over long time scales. Considering this, the theoretical best model would correspond to a mixed strategy: several taxa from several kingdoms show an alternation from sexual to asexual mode [10].

Most reef-building corals present various mixed strategies for reproduction (reviewed in [11]). Generally, for marine benthic organisms and particularly for scleractinian corals, sexual reproduction is achieved by either spawning gametes with external fertilization (spawners), or by internal fertilization and brooding larvae inside the coral polyp (brooders) [11]. In both cases, the formed planulae disperse, settle and metamorphose to form new colonies. Additionally, some corals have been described to reproduce asexually by vegetative propagation in different ways: fragmentation [12], budding or polyp expulsion [13], or release of asexual larvae, which has been evidenced in few scleractinians such as *Pocillopora* species (*e*.*g*., [14, 15]) and *Tubastrea* species [16]. Indeed, parthenogenetic larvae production has been found to be an alternate reproductive mode for corals when sexual reproduction leads to high failure in fertilization rates in cases of sperm limitation [17].

The common branching coral *P*. *damicornis* (Linnaeus, 1758) is found in lagoons from Red Sea, Indian and Pacific Oceans in shallow habitats [18]. It has been described to present (1) a huge morphological plasticity linked to environmental conditions and depth [19] and (2) sexual and asexual reproduction modes with abilities to be either brooder or spawner [20]. Parthenogenetic larvae production in *P*. *damicornis* has been reported for thirty years using different genetic tools: isozymes [14], allozymes (*e*.*g*., [15]) or microsatellites (*e*.*g*., [21]). However, these findings are to be taken with a grain of salt as the taxonomy of *Pocillopora* species has been recently revised using the mitochondrial Open Reading Frame (ORF) sequences (840 bp), showing five distinct lineages (*α*, *β*, *γ*, *δ* and *ε*) under the *P*. *damicornis* designation [22]. Concerning our study, we focused on *P*. *damicornis* type *β*, also designated as *P*. *acuta* (*sensu* [23]), *Pocillopora* type 5 [24], *P*. *damicornis* type F [25] and *Pocillopora* Clade Ia [26]. Without judging these different designations, we chose to use the name *P*. *damicornis* type *β* (referred to *P*. *damicornis β* hereafter for lightening the writing) to refer to the lineage identity herein under study, as it seems to us the more explicit considering the knowledge on species delimitation in this genus to date. Thus, former studies dealing with *P*. *damicornis sensu lato* should be reconsidered in the light of these new taxonomic insights, as molecular identification of colonies is revealed as an absolute necessity. As an example, Pinzón et al [27] revealed high clonal propagation over small distances (*i*.*e*., ≤ 10 km) in colonies from ORF type 1 that the authors designated as *P*. *damicornis*. Nevertheless, in Pinzón et al [24], the authors showed that type 1 colonies form a clade corresponding to various morphs (*damicornis*, *verrucosa*, *elegans*, *meandrina*, *capitata*, *eydouxi*), which Schmidt-Roach et al [23] attributed to *P*. *eydouxi/meandrina* complex. Similarly, Gorospe and Karl [28] studied clonal propagation in *P*. *damicornis* in Hawaii, showing an over-representation of one clone at a scale of 1.5 km: from their sampling, they found two lineages (one represented by 98.8% of their samples), identified *a posteriori* as *P*. *damicornis* lineages *α*, *β* or type 6 (*sensu* [24]) [29], but the authors did not indicate which lineage was the most represented in their first study. Likewise, in Combosch and Vollmer [21], it seems that two lineages were present as the planulation date was different from one colony to another. Indeed, some spawned at the new moon and others at the full moon, indicating that they may be from lineages *β* and α, respectively (see discussion in [22]). Concerning the earlier studies focusing on *P*. *damicornis* clonal propagation [14, 15, 30–33], they did not provide the ORF sequences nor indications about planulation date, so it is not possible to determine which *P*. *damicornis* lineage they dealt with and to extrapolate about clonal propagation of *P*. *damicornis β*. Contrary to all the studies cited above, few studies presented no doubt about *P*. *damicornis* identification. Indeed, the studied colonies from Souter et al [25] were assessed *a posteriori* to *P*. *damicornis β*. This latter found clonal propagation over 60 km on the East Coast of Africa. Moreover, in Polynesia, high clonal propagation was found in *P*. *damicornis β* and clones were found to be extended over very large distances (*ca*. 200 km; [34]). Conversely, in the Great Barrier Reef, Torda et al [35] found very short distance of clonal propagation (not more than 230 m) in *P*. *damicornis β*.

Given the new taxonomic insights in the *Pocillopora* genus and the few studies for which *P*. *damicornis* lineages were clearly identified,the aim of our study was to investigate, in Reunion Island (Western Indian Ocean), the clonal propagation and population structure in the coral *P*. *damicornis β*. For this purpose, we used 13 microsatellite loci to identify genetically sampled colonies and we chose four study sites presenting contrasted environmental conditions (hydrodynamics, temperature, tourism activities,…) along the west coast of Reunion Island to assess the relative proportions of each reproductive modes in this species and the distance of clonal propagation at the island scale.

## Materials and Methods

### Sampling design

**Fig 1:**
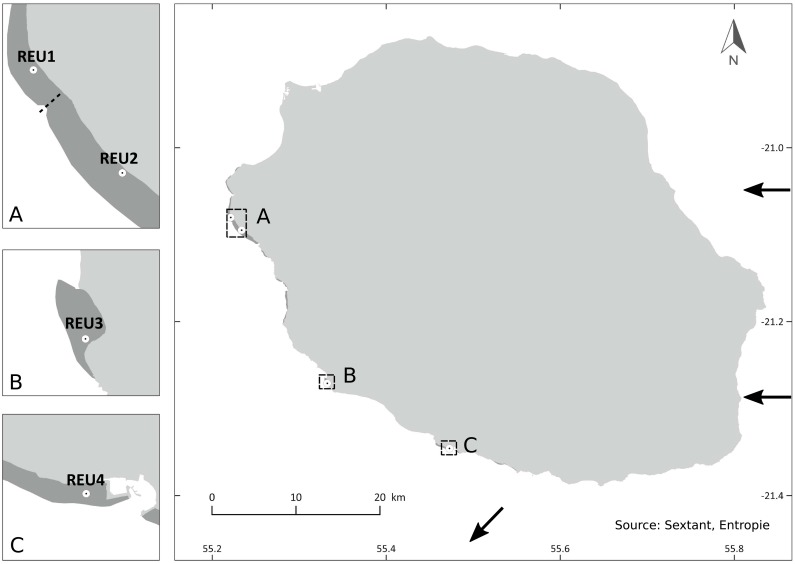
Map of the sampling sites of *Pocillopora damicornis* type *β* in Reunion Island. Lagoons are represented in dark grey. The channel between REU1 and REU2 is symbolized with a dash line and the direction of the South Equatorial Current around Reunion Island is symbolized with black arrows (from [37]). (Open access data: http://sextant.ifremer.fr/fr/web/ocean_indien/geoportail/sextant#/metadata/f3587329-b5b7-4ee1-822d-5cda9cef4d09).

Colonies of *P*. *damicornis β* were sampled in lagoons, at four sites located along the west coast of Reunion Island (Fig 1), situated in the South Western Indian Ocean, 700 km east of Madagascar. These sites, chosen for their contrasted environmental conditions and differences in coral density, were accessible by snorkeling in one meter depth and were named, from North to South, REU1, -2, -3, -4. REU1 and REU2 are located on the north part of the west coast of Reunion Island, where the lagoon is at its widest (approximately 500 m) and where touristic activities induce disturbances on the reef. REU4 and in lesser proportions REU3, the southernmost sites, are exposed to strong swells and waves during the southern winter and are less frequented, contrasting with REU1 and REU2 sites which are located in less exposed reefs with weaker hydrodynamics [36].

REU1 (S21°04'56.49", E55°13'22.19") and REU2 (S21°05'47.94'', E55°14'01.68"), separated by a shallow channel, are located approximately 2 km apart in the lagoon complex of La Saline-L’Ermitage. REU3 (S21°16'15.17'', E55°19'56.31") and REU4 (S21°20'44.68'', E55°28'21.66") are located in separated lagoons, REU3 approximately 25 km south of REU1-REU2 and REU4 approximately 40 km south of REU1-REU2 (Fig 1).

#### Exhaustive sampling

In REU1, colonies were distributed along with *Acropora* patches separated by sand strips. As the colonies distribution was not uniform, an exhaustive sampling was set up. We sampled all colonies (*N*_*total*_ = 183) found in a 12 m radius circle centered on a patch and recorded the X-Y coordinates using two tape measures placed perpendicularly to materialize the axes. A third tape measure combined to a 1 m x 1 m quadrat was used to move along both axes.

#### Random sampling

**Fig 2:**
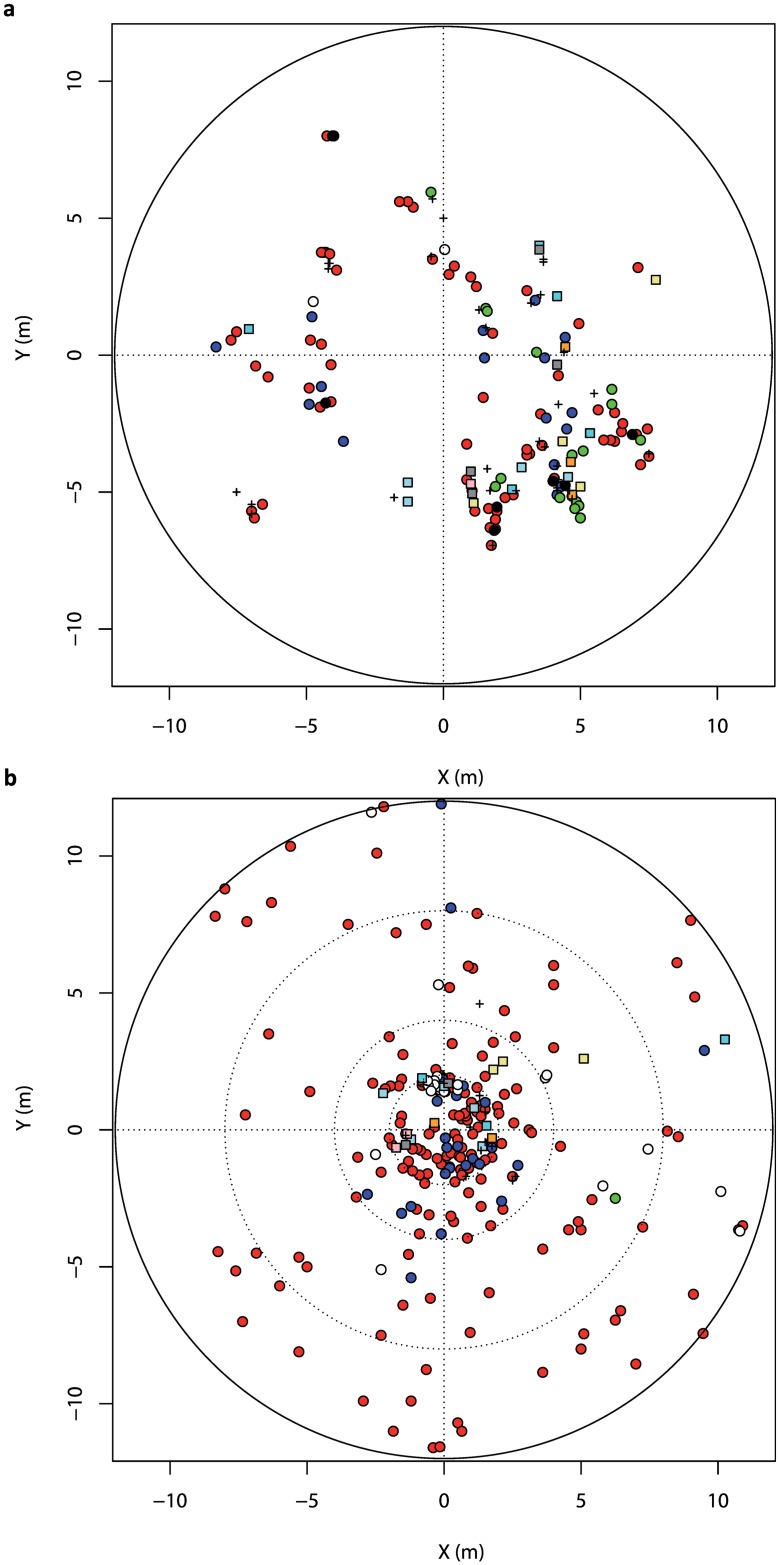
Spatial representation of *Pocillopora damicornis* type *β* colonies sampled in (a) REU1 (*N* = 158) and (b) REU2 (*N* = 264). Each symbol represents a colony. Multi-locus genotypes (MLG) occurring only once are represented with “+”. Colonies sharing the same MLG are represented with the same color and each repeated MLG is represented by a different color. MLGs shared by both sites are represented with circles (MLG01: red, MLG02: blue, MLG03: white, MLG04: green, MLG05: black) and MLGs specific to a unique site with squares.

In REU2, as colonies were distributed uniformly on the substrate and easily accessible without damaging the reef, colonies were sampled following a random sampling method using four nested circles of 2, 4, 8 and 12 m radius, respectively (*N*_*total*_ = 297; Fig 2). Within each strip, 50 colonies were randomly collected, except in the central circle (2 m radius) where an exhaustive sampling was conducted to study micro-scale clone dispersion (from 0 to 12 m). Practically, graphic coordinates had been previously randomly generated in the different circles. Then, in the field, a colony situated approximately in the middle of the *P*. *damicornis β* patch was selected as origin of the plan. The same plan as for REU1 was set up for recording the coordinates.

#### Haphazard sampling

For REU3 and REU4, a random sampling scheme was unfortunately not applicable. Indeed, in REU3, the high coral cover combined with low depth prevented us from setting up the same kind of sampling scheme without damaging the reef. Also, in REU4, *P*. *damicornis β* colonies were too scattered to sample enough colonies in a 12 m radius circle. Thus, colonies from these two sites were sampled haphazardly while snorkeling, without reporting coordinates (*N*_*total*_ = 50 and 57, respectively).

All colonies were photographed and their color reported (pink or cream). A small fragment (1–3 cm) was removed from each colony, placed into a numbered zip-lock bag, fixed in 90% ethanol and stored at room temperature.

Hereafter, population refers to all the colonies sampled at one sampling site.

### DNA extraction, sequencing, and microsatellite genotyping

From the 587 sampled colonies, DNA was extracted using DNeasy Blood & Tissue kit (Qiagen^™^). Colonies were genotyped using 13 microsatellite loci [28, 38–41] (S1 Appendix). Forward primers were indirectly fluorochrome labelled (6-FAM, VIC, NED, PET) by adding a universal M13 tail at the 5'-end and were multiplexed post-PCR in three panels (S1 Appendix). Each amplification reaction was performed as in Postaire et al [42]. PCR products were genotyped using an ABI 3730 genetic analyser (Applied Biosystems) and allelic sizes were determined with GeneMapper V4.0 (Applied Biosystems) using an internal size standard (Genescan LIZ-500, Applied Biosystems). Individuals showing peak profiles either faint or with more than two peaks were processed again and, when remaining ambiguous, designated as missing data to avoid dealing with more than two alleles (resulting either from non-specific amplifications or somatic mutation). However, it should be noted that keeping the third allele, particularly for Pd3-EF65, did not interfere with multi-locus genotypes (MLG) identification (PG pers. obs.; [28]). Finally, 519 colonies without missing data were kept for further analyses. Because colonies were sampled based on their *corallum* macromorphology, *P*. *damicornis β* lineage identity was verified by amplifying a 840 bp fragment of the mitochondrial open reading frame (ORF) region with the FATP6.1 and the RORF primers and using the same conditions as described in Flot and Tillier [43], for 108 colonies selected *a posteriori* based on their MLG at microsatellite loci (see below). Amplifications were sent for sequencing to Genoscreen (Lille, France) and edited with Geneious R7 [44].

### Analysis of clonal structure

The occurrence of identical MLGs among the sampled colonies was assessed using GenClone V2.0 [45]. The probability of obtaining the same MLG more than once from distinct random reproductive events was further estimated using *P*_*SEX*_ (*F*_*IS*_), which considers possible departure from Hardy-Weinberg equilibrium in order to obtain a less biased estimator [46]. Colonies sharing the same MLG resulting from a unique reproductive event (one unique zygote) were considered as belonging to the same clone. Since somatic mutations [47] or scoring errors may overestimate the true number of clones, pairwise genetic distances were calculated among all colonies using GenoType [48] based on mutational steps under the stepwise mutational model. A threshold in the genetic distance distribution was determined under which distinct MLGs were considered as the same multi-locus lineage or MLL (*i*.*e*., clones genetically close, *sensu* [45]).

For each population, the clonal richness *R* was estimated [49], as well as the genotypic diversity *G*, estimated as *G*_*O*_/*G*_*E*_, where *G*_*O*_ is the observed genotypic diversity as described by [50] and *G*_*E*_ is the expected genotypic diversity as described in Baums et al [51]. *G* reflects the reproduction mode, varying from 1 in sexual populations to 0 in clonal ones. The average number of alleles per locus (allelic richness; *Â*) was estimated with the truncated dataset (*i*.*e*., keeping one representative per MLG per population) with FSTAT V2.9.3 [52]. Moreover, to assess the relevance of the loci used (number and variability), we estimated the probability that two randomly sampled MLGs share the exact same alleles over all loci just by chance rather than being the result of asexual reproduction (probability of identity *P*_*ID*_, [53]) with GIMLET V1.3.3 [54].

With the aim to assess clonal heterogeneity and evenness of all our populations, the parameter *β* of the Pareto distribution and the Simpson’s evenness (ED*) were estimated with GenClone V2.0 [45] within each population. Moreover, in order to investigate the spatial distribution of genotypes in each population for which spatial coordinates were available (REU1 and REU2), the aggregation index (*Ac*) and edge effect (*Ee*) were estimated and their significance tested with 10^3^ permutations in GenClone V2.0 [45]. Moran’s spatial autocorrelation index (*I*) was further estimated with SPAGeDi V1.5 [55] to assess whether genetic relatedness is related to geographic distance between colonies.

### Population genetic differentiation

The genotypic disequilibrium among pairs of loci was assessed with FSTAT V2.9.3 [52], on the truncated dataset. The presence of null allele was tested using Micro-Checker V2.2.3 [56]. Departures from Hardy-Weinberg equilibrium (HWE) for each population (*F*_*IS*_) were estimated using GENETIX software V4.05.2 [57] with 10^4^ permutations.

Population differentiation indices *F*_*ST*_ [58] and *D*_*est*_ [59] were estimated both on the entire dataset (*i*.*e*., keeping all individuals) and on the truncated one. Indeed, as some genotypes might present a higher fitness than others, keeping all the colonies sharing these genotypes might bias population differentiation estimation, as well as keeping only one representative per genotype. Thus, fitness of each genotype is taken into account when all repetitions of each MLG are kept while the truncated dataset considers all fitnesses equally (as in [60]). For both cases, hypotheses are probably wrong and the real differentiation between populations is likely intermediate between both scenarii.

Additionally, to determine the most likely number of genetically homogenous clusters (K) within our dataset, a Bayesian clustering analysis was performed using InStruct [61], which considers partial self-fertilization or inbreeding. Conditions were set to 10^6^ chain length after a burn-in of 10^5^ and 10 chains were run for each K varying from 1 to 10. The analysis was performed both on the entire and the truncated datasets for comparisons. The optimal K was chosen based on lower values of the Deviance Information Criterion (DIC, [62]) and the Evanno’s method [63]. For both cases, graphs were drawn using Distruct V1.1 [64] after combining the different replicates for each K in Clumpp V1.1.2 [65]. Additionally, in order to explore the diversity structure without any prior hypothesis regarding our populations (HWE, LD), we performed a Principal Components Analysis (PCA) based on a dissimilarity matrix using Dice distance between MLGs with Darwin V6.0.9 [66] and constructed a network based on shared allele distance using EDENetwork V2.18 [67].

## Results

All 13 loci used were polymorphic in each population except locus PV7 in REU2. The allelic richness ranged from 2.46 for Pd04 to 6.07 for Poc40 (S1 Appendix). The combination and high variability of the 13 microsatellite loci used here resulted in a very low probability (*P*_*ID*_ = 1.337×10^−5^) that two colonies sharing the same MLG were identical by chance, so that shared MLGs were considered to be the result of asexual reproduction. The use of these 13 loci therefore allows uncovering the entire genotypic diversity of the dataset.

Over the 519 genotypes without missing data, 108 distinct MLGs were detected. For one representative of each MLG, the mitochondrial ORF was sequenced (840 bp; accession numbers from KT879932 to KT880039) and compared to GenBank sequences identified as *P*. *damicornis* type *β*, *P*. *damicornis* type *α*, *P*. *damicornis* type *σ*, *P*. *damicornis* type *ε*, *P*. *verrucosa*, *P*. *meandrina* and *P*. *eydouxi* (JX625045 to JX625077, JX983183 to JX983186 and JX985610 to JX985613; [22, 23]. Over our 108 sequenced colonies, nine were identified as belonging to the *P*. *meandrina/eydouxi* complex (one from REU1 and five from REU4) and *P*. *verrucosa* (one from REU1 and two from REU4) and were removed from the dataset. Finally, for further analyses, were kept 510 *P*. *damicornis β* colonies (all sharing the exact same mtDNA haplotype), corresponding to 99 different MLGs.

The hypothesis of multiple reproduction events being responsible for the presence of several colonies sharing the same MLG was rejected in all cases (all *P*_*SEX*_(*F*_*IS*_) < 1.24×10^−5^), indicating that colonies with identical MLG belonged to the same clone. Moreover, the first gap in the distribution of pairwise allelic differences among the 99 MLGs was found between 0 and two mutation steps. Thus, to compensate for genotyping errors and possible somatic mutations, we chose a threshold of one mutation step to differentiate MLLs, as found in other studies (*e*.*g*., [68]). Since pairwise MLGs differed by at least two mutation steps, all distinct MLGs were considered as distinct MLLs. To avoid redundancy, we further kept the term MLG rather than MLL for the 99 different MLGs found among our sampling.

**Fig 3:**
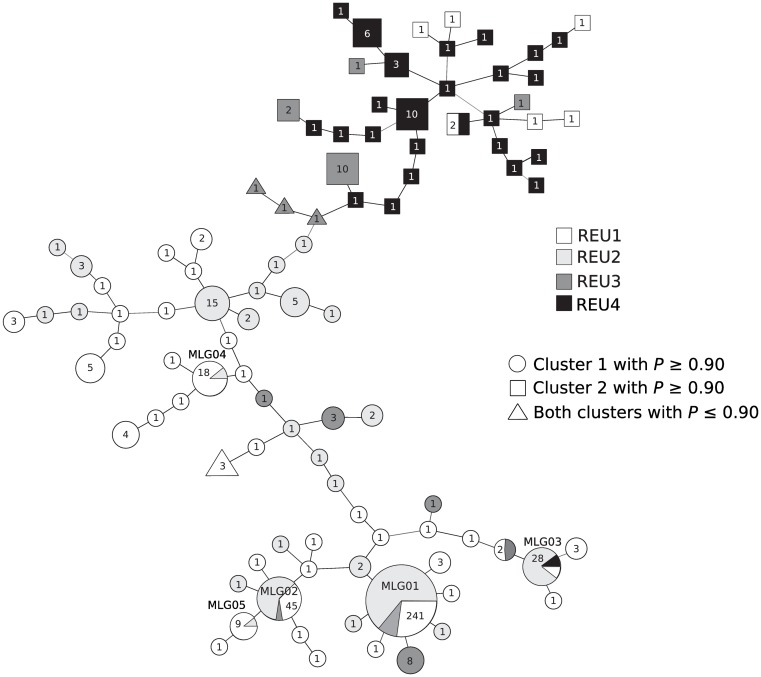
Network of multi-locus genotypes (MLG) based on shared allele distance. The number of colonies sharing each MLG is indicated inside each node and the geographic origin of the colonies is represented by colors. The node shape represents the result of the clustering analysis: circles, MLGs assigned to cluster 1 (see Fig 4) with a probability ≥ 0.90; squares, MLGs assigned to cluster 2 (see Fig 4) with a probability ≥ 0.90; triangles, MLGs assigned to one cluster or the other with a probability ≤ 0.90.

Over the 99 MLGs, 27 occurred more than once, being shared by several colonies within sites, as well as between sites for seven of these MLGs (Fig 3). Interestingly, one MLG (MLG01) was over-represented (47% of the overall colonies sampled), especially in REU1 and REU2 presenting 82% and 81% of the sampled colonies, respectively (Fig 2). Furthermore, three MLGs (including the two most highly common ones, MLG01 and MLG02; Fig 3) were observed both in REU1 and REU2, separated by 2 km. More surprisingly, MLGs were found in much more distant sites, between REU1 and REU3 (MLG01 and MLG02; Fig 3) approximately 25 km apart and between REU1 and REU4 (MLG03), located approximately 40 km apart. Furthermore, it is noteworthy that colonies sharing a same MLG did not always display the same colour (pink or cream).

Within populations, the clonal richness varied from 0.095 in REU2 to 0.596 in REU4 (Table 1). Additionally, the genotypic diversity *G* was low for all populations and ranged from 0.006 in REU2 to 0.264 in REU4. Concerning Simpson’s evenness index and the parameter *β*, the lowest values were found for REU2 (very low slope; S2 Appendix), revealing the presence of few MLGs but highly and non-evenly repeated (Table 1, S2 Appendix). Conversely, in REU4, there were numerous MLGs repeated a few times and more evenly (steepest slope for Pareto distribution; S2 Appendix). REU1 and REU3 presented an intermediate slope between the two others.

**Table 1:** Summary statistics for the four *Pocillopora damicornis* type *β* populations.

Population	REU1	REU2	REU3	REU4
*N*_*total*_	183	297	50	57
*N*	158	264	42	46
*N*_*MLG*_	44	25	13	27
*R*	0.274	0.095	0.293	0.596
*G*	0.016	0.006	0.129	0.264
ED*	0.681	0.514	0.755	0.706
*β*	0.212	0.085	0.375	0.625
*Ac*	0.109***	0.162***	-	-
*Ee*	-0.476	-0.472	-	-
Moran’s *I*	0.000	-0.024	-	-
*F*_*IS entire*_	-0.058**	-0.262***	0.055^NS^	0.182***
*F*_*IS truncated*_	0.110**	-0.037^NS^	0.213***	0.279***
*H*_*E*_	0.485 (0.182)	0.428 (0.215)	0.559 (0.171)	0.586 (0.141)
*H*_*O*_	0.432 (0.307)	0.443 (0.329)	0.444 (0.252)	0.424 (0.292)

*N*_*total*_: number of colonies sampled*; N*: number of colonies presenting no missing data in their multi-locus genotypes (MLG); *N*_*MLG*_: number of MLG; *R*: clonal richness; *G*: genotypic diversity; ED*: Simpson’s evenness; *β*: parameter of the Pareto distribution (-1*regression slope); *Ac*: aggregation index; *Ee*: edge effect; Moran’s *I*: spatial autocorrelation index, *F*_*IS entire*_: values are estimated on the entire dataset; *F*_*IS truncated*_: values are estimated on the truncated dataset (one representative per MLG and per population); *H*_*E*_ (s.d.) and *H*_*O*_ (s.d.): expected and observed heterozygosities respectively, values are estimated using the truncated dataset. ^NS^: non-significant;

** *P*<0.01;

*** *P*<0.001.

Spatial analysis examining MLG distribution were performed within REU1 and REU2 sites. The aggregation coefficient was significantly positive in both sites (*Ac* = 0.109, *P* = 0.004 and *Ac* = 0.162, *P* < 10^−3^, respectively), suggesting that colonies belonging to the same clone were spatially closer than colonies belonging to different clones. However, spatial autocorrelation analysis did not show a significantly higher relatedness among geographically close MLGs as compared to distant ones (REU1: *I* = 0.000, *P* = 1.000; REU2: *I* = -0.024, *P* = 0.611). Also, no significant edge effect was detected in any site (REU1: *Ee* = -0.018, *P* = 0.620; REU2: *Ee* = -0.472, *P* = 0.991), suggesting that new MLGs were not especially detected on the edge of the sampling zone (Fig 2).

Keeping only one representative per MLG, no evidence of linkage disequilibrium was detected between loci; none of the 78 tests were significant at the 0.05 level after correction for multiple testing (Bonferroni). Then, all loci were interpreted as independent loci. Two loci (Pd2-001 and Poc40) evidenced null alleles in all populations; they were kept for analysis of clonal diversity as they allowed discriminating different MLGs, but removed for the following population structure analyses. *F*_*IS*_ values (Table 1) estimated on the entire dataset (*N* = 510) were either significantly negative for two populations REU1 and REU2 (-0.058, *P* < 10^−2^; -0.262, *P* < 10^−3^, respectively) or significantly positive for REU4 (0.182; *P* < 10^−3^), or not significantly different from zero for REU3. When estimations were performed on the truncated dataset (*i*.*e*., one representative per MLG and per population kept; *N* = 109), *F*_*IS*_ values increased and became significantly positive except for REU2 (-0.037, *P* > 0.05; Table 1).

When including all colonies, each population was highly differentiated from the others, with pairwise *F*_*ST*_ estimates ranging from 0.016 (*P* < 10^−3^) between REU1 and REU2, to 0.428 (*P* < 10^−3^) between REU2 and REU4 (Table 2). Noteworthy, *D*_*est*_ estimates were of the same order of magnitude, ranging from 0.012 (*P* < 10^−3^) between REU1 and REU2, to 0.376 (*P* < 10^−3^) between REU2 and REU4 (Table 2). These results remained nearly identical when *F*_*ST*_ were estimated on the truncated dataset, though they were approximately reduced by a 1.5 order (Table 2).

**Table 2:** Genetic differentiation between *Pocillopora damicornis* type *β* populations estimated with Weir and Cockerham’s *F*_*ST*_ [58] and with Jost’s *D*_*est*_ (in parentheses, [59]).

	**REU1**	**REU2**	**REU3**	**REU4**
**REU1**	-	0.011 (0.010)	**0.068 (0.092)**	**0.202 (0.293)**
**REU2**	**0.016 (0.012)**	-	**0.094 (0.088)**	**0.262 (0.339)**
**REU3**	**0.090 (0.067)**	**0.126 (0.066)**	-	**0.081 (0.185)**
**REU4**	**0.348 (0.349)**	**0.428 (0.376)**	**0.181 (0.250)**	-

Values in the lower matrix are estimated on the entire data set; values in the upper matrix are estimated on the truncated data set. Values in bold are significant (*P* < 0.05).

**Fig 4:**
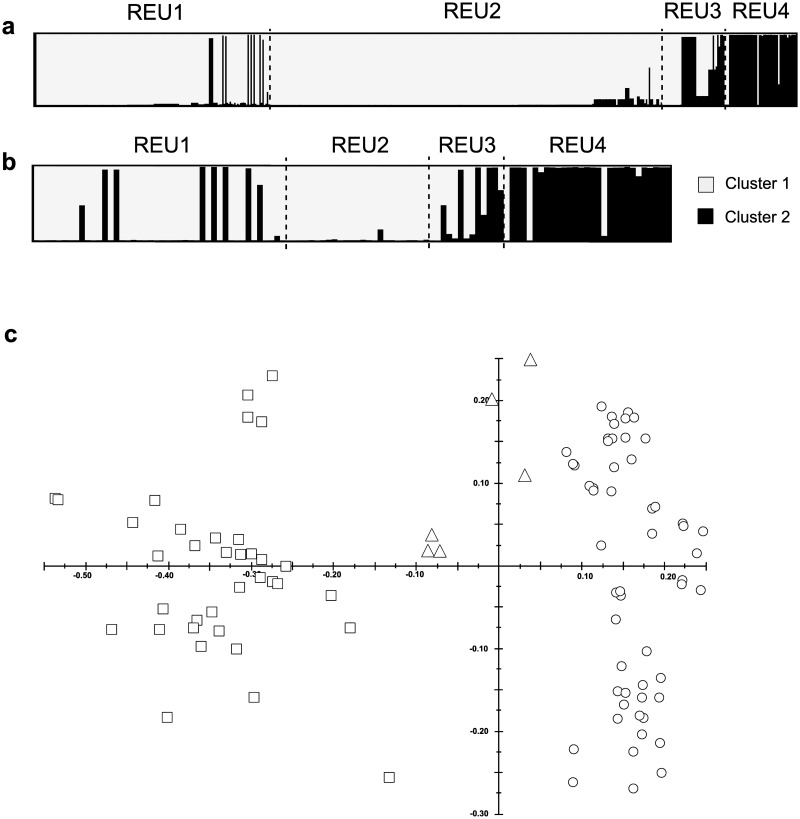
**Results of the clustering analysis for K = 2,** (a) for the entire dataset (*N* = 510) and (b) for the truncated dataset containing one representative per multi-locus genotype and per site (*N* = 109). For each plot, populations are separated with dash lines. (c) Principal Component Analysis estimated on the truncated dataset (based on 13 microsatellites). Axes 1 (horizontal) and 2 (vertical) represent 31.56% and 10.88% of the total variance, respectively. Symbols are the same than in Fig 3.

Results of the clustering analyses led to similar results according to the DIC method or the Evanno’s one (S3 Appendix). Both methods gave an optimal K of 2 for both datasets, the MLGs being assigned to the same cluster whatever the dataset used, naturally implying that colonies sharing the same MLG were assigned to the same cluster (Fig 4a and 4b). The PCA (based on the information from either 13 or 11 microsatellites), when keeping the two first axes, gave the same partition in two groups corresponding to the two clusters identified by the Bayesian analysis (Fig 4c). Thus, with few individual exceptions, the two clusters segregated the northern populations (REU1 and REU2) from the southernmost population (REU4) (Fig 4). Indeed, the two northernmost populations (REU1 and REU2) were almost exclusively composed by colonies assigned to cluster 1, and nearly all colonies of REU4 were assigned to cluster 2. REU3, located nearly half-distance between REU2 and REU4, was characterized by a mix of both clusters: over 13 MLGs, six were assigned to the northern cluster (cluster 1; probability ≥ 0.90), four to the southern cluster (cluster 2; probability ≥ 0.90) and three for which the probability to be assigned to one or the other cluster was less than 0.68 (Figs 3 and 4b).

## Discussion

Our results revealed a high proportion of repeated MLGs per reef, on all studied sites, suggesting high rate of asexual reproduction in *Pocillopora damicornis β* from Reunion Island reefs. One superclone (representing 47% of all studied colonies) was even found in all sites, suggesting that long-distance dispersal (40 km) of some clones is not uncommon. Despite, this long dispersal, a small scale genetic differentiation was revealed between Northern and Southern populations.

The use of a random sampling scheme is theoretically the most pertinent way to assess clonality [46] as every possible sampling unit would present equal probability of being included in the population. It also allows to minimize bias in estimation of diversity and richness indices [46]. However, it is the most difficult to proceed practically and this scheme could not be applied in all our four study sites, but only in REU2. Beyond the sampling scheme, the sampling effort is also determinant to reduce bias in index estimations [46], which was taken into account in our sampling scheme. Despite all precautions, the exhaustive sampling only in the 2 m radius central circle in REU2, as well as the colony patchiness in REU1 may have biased our estimated indices. From another angle, edge effect and spatial autocorrelation indices indicated that new MLGs were not especially detected on the edge of the sampling scheme and were randomly distributed, which indicates that the bias is minimized.

### Superclone expansion

In the lagoon of La Saline–L’Ermitage complex where REU1 and REU2 sites are located, we found the co-existence of one “giant” clone (represented by a large number of colonies sharing MLG01) and several less represented ones, with 71% of our sampled colonies presenting one of the seven most abundant MLGs (out of 99) (as revealed by the slow values of the parameter β of the Pareto distribution and the Simpson index). Such result of over-representation of few clones has already been observed in *P*. *damicornis sensu lato* on a patch reef in Kane’ohe Bay, O’ahu (Hawaii) [28], where 70% of the sampled colonies represented only 10% of the MLGs (7 out of 78). This over-representation of one genotype could be the result of asexually produced larvae [21, 31]. Nevertheless, Torda et al [69], studying *P*. *damicornis* type *β* on the Great Barrier Reef, found that clonal larvae only represented 7% of the brooded larvae. Moreover, considering that *P*. *damicornis β* presents a rather thin morphology combined with a breakable skeleton, the high dominance of one genotype in this northernmost lagoon is likely the result of fragmentation, mainly induced by the exposition to waves and touristic activities (confirmed by the significantly positive aggregation coefficient in REU1 and REU2 sites). Also, no significant edge effect was detected in any site (REU1: *Ee* = -0.018, *P* = 0.620; REU2: *Ee* = -0.472, *P* = 0.991), suggesting that new MLGs were not especially detected on the edge of the sampling zone (Fig 2). Indeed, the very low depth (approx. 1 m) of this lagoon makes it highly frequented by bathers and snorkelers and we observed some living fragments not yet fixed on the substratum. Additionally, the occurrence of clones may as well result from polyp bail-out [13], a well-known phenomenon by aquarium hobbyists dealing with *P*. *damicornis sensu lato* but poorly documented.

The expansion of one superclone might result from a combined effect of asexual propagation with high fitness related to particular environmental conditions [28, 70]. Such phenomena start to be investigated in corals with some studies reporting superclone dominance in different species, like the monoclonal population of *Acropora palmata* in a Caribbean reef [71] or for *P*. *damicornis sensu lato* in Hawaii [28] and for *Pocillopora* type 1 in the Tropical Eastern Pacific [27]: in these cases, the dominant clone is likely well-adapted to local environmental conditions and its expansion reduces the genotypic diversity on reefs. Indeed, this large superclone dominance could lead to a negative fate of the population as illustrated in two stable reefs from Florida Keys which presented allelic and genotypic losses over four years in highly clonal populations of *A*. *palmata* [72]. Moreover, in case of perturbation (*e*.*g*., warming, pollution or pathogen emergence), the over-represented genotype might not be longer well-adapted to the new conditions and the population could collapse, while a population presenting a higher genotypic diversity is more likely to resist such a perturbation, as it presents a higher probability that one or some of its genotypes could cope [73].

The higher genotypic diversity and clonal richness found in REU4, the southernmost site, may be due to its exposition to stronger swells and waves during the southern winter, contrasting with REU1 and REU2 sites which are located in less exposed reefs with weaker hydrodynamics [36]. Indeed, genotypic diversity is found to be related to high disturbance history [74]. In REU4, colonies were more compact and the branches shorter and tighter than those from the other sites, resulting in a less breakable skeleton and lower clonal propagation. The presence of these particular *Pocillopora* morphotypes explains also the misidentification with *P*. *verrucosa* and *P*. *eydouxi* that occurred during sampling.

### Long-distance dispersal

Around the island, we found seven MLGs shared by several populations, suggesting a strong clonal propagation. The two furthermost populations sharing identical MLGs were located nearly 40 km apart (REU1 and REU4). To date, two studies found a similar result, with clones shared among populations 40 km apart [25] and 200 km apart [34]. As the estimated probability that two colonies sharing the same MLG were identical by chance (*i*.*e*., *P*_*ID*_) is less than 10^−4^ (recommended threshold when using more than 10 loci presenting high allelic richness [53]), it is most likely that the identical MLGs found result from asexual propagation. Moreover, fragmentation, while being an obvious explanation at the intra-reef levels, becomes a weak hypothesis to explain the occurrence of clones over such large distances. Indeed, over higher distances, it seems unlikely that coral fragments could break off from one reef, drift for several kilometres and be fixed again on another reef, especially in our case where the two reefs in question are separated by high water depths and “no-reef” zones. Polyp bail-out seems also unlikely at this scale, for the same reasons exposed previously and because of their likely negative buoyancy and limited power of mobility, though this hypothesis cannot be ruled out as free polyps of *Seriatopora hystrix* can leave up to 9 days before re-attaching to the bottom [13].

An alternative hypothesis is that distant clones could be the result of parthenogenetic larvae [21, 31] which appears to be a more solid explanation in our case. Indeed, it has been hypothesized that asexually produced larvae (larger and better-provisioned) may be more effective to disperse than sexual progenies issued from broadcast spawning [75], these latter tending to settle locally [76]. A mixed reproduction mode has moreover already been shown for *P*. *damicornis sensu lato* [14, 21], with parthenogenetic larvae production dealing with sperm limitation, a general issue for broadcast sperm-dependent marine animals [17]. Anyhow, neither parthenogenetic larvae nor released polyps hypotheses can be verified directly in our study. Additional experiments in controlled environments are needed to fully conclude on their role in clonal propagation.

### Two highly differentiated lineages

The two genetically distinct clusters revealed among all sampled colonies by the Bayesian assignment test more or less differentiate northern from southern populations, whatever the dataset used (truncated and entire datasets). We acknowledge that keeping all individuals (entire dataset) for the genetic structuring analysis may introduce a bias, but no more than keeping one representative per MLG (truncated dataset). Nonetheless, our analyses using both datasets gave exactly the same individual assignment suggesting that the pattern we observed here is robust. Despite the large genetic divergence among the two clusters (*F*_*ST*_ = 0.209), all colonies included in this analysis shared the exact same mtDNA ORF haplotype, assigned to *P*. *damicornis β*. Pairwise *F*_*ST*_ estimates involving comparisons between northern and southern populations were consequently high, whatever the dataset used. Such estimates are in the order of magnitude of what could be expected between different species using these markers, like for example between two sympatric *Pocillopora* types, based on seven microsatellite loci (type 1 and type 5; *F*_*ST*_ = 0.346; [24]). Other studies conducted on *P*. *damicornis sensu lato* showed similar high levels of genetic differentiation, such as found among Pacific populations (*e*.*g*., [30]), or in the Gulf of Panama between populations separated by approximately 15 km (*F*_*ST*_ > 0.22; [76]), but in these examples, high values could result from a mix of different lineages of *P*. *damicornis sensu lato* in different proportions. All in all, the high *F*_*ST*_ estimates reflect the allele frequencies variance between two genetically distant clonal lineages (one in the North and one in the South), each composed of several genetically close MLGs (Fig 3).

Altogether, our results showed on one side, the detection of shared clones among distant sites indicates that larvae dispersal among reefs in not uncommon, while on the other side, the significant genetic structure among the four sites suggests that gene flows appeared limited along the west coast of Reunion Island. Our result contrasts with the genetic structure to be expected from a species with a larval survivorship estimated to reach 100 days [77, 78]. Indeed, with such long larval duration, one would expect to find limited population genetic differentiation due to potential high larval dispersal abilities [79, 80], which is not what we found, nor what was observed in a previous study conducted in the same region, South Western Indian Ocean, and on the same lineage, *P*. *damicornis β* ([24]; named *Pocillopora* type 5 therein). Additionally, at very small scale (approximately 1 km), our results are concordant with another study conducted in Hawaii [28] revealing no genetic differentiation, as between REU1 an REU2, separated by 2 km.

Apart from geographic distances, various factors are known to modify the predicted dispersal abilities, such as ocean currents, planktonic environment or larval behaviour and settlement ability [81]. In the Indian Ocean, the South Equatorial Current (SEC), circulating from east to west between 7–8°S and 23–25°S and bathing the South coast of Reunion Island [82] is likely to create a geographic barrier between the northern and southern populations, showing different hydrodynamic conditions. Thus the fact that REU4, the southernmost population, appeared as a group genetically distinct from the three other populations could therefore result (1) from a particular connection of the southern part of Reunion Island with other reefs from Mauritius or Rodrigues through the SEC and (2) from the flushing out of larvae broadcasted from REU4 to the west (towards Madagascar), without going to the north of Reunion Island. Besides, on the west coast of Reunion Island (REU1, REU2 and REU3), oceanic currents are complex, due to local scale eddies created by the SEC residues [37]. Then, larvae from elsewhere might have some issues to reach the northern reefs and larvae produced on the west coast might be retained by local currents.

On the other hand, this structuring pattern could also reflect the presence of two different lineages partially reproductively isolated in the *P*. *damicornis β*, as recently highlighted in *Acropora* corals from Western Australia [83], that may result from asynchronous spawning. Reviewing studies focusing on *P*. *damicornis sensu lato* reproduction in light of new taxonomic insights Schmidt-Roach et al [84] concluded that *P*. *damicornis β* broods its larvae and releases them after the new moon, produces parthenogenetic larvae and spawns male gametes. To our knowledge, asynchronous release of gametes or larvae has not been reported yet for *P*. *damicornis β*. However, two *P*. *damicornis* populations of the Great Barrier Reef located 13 km apart, characterised *a posteriori* as type *α* by Schmidt-Roach et al [22], showed differences in larvae release dates: from October to November in One Tree Island [31] and from September to February in Heron Island [85] underlying the possibility of asynchronous spawning events over small spatial scales.

In Reunion Island, although the spawning period of *P*. *damicornis β* is unknown, the hypothesis of asynchronous spawning cannot be ruled out to explain the presence of these two genetically distinct clusters whose distributions overlap in REU3. Indeed, one could imagine that, considering the short gamete lifespan in the water column in scleractinians (*e*.*g*., < 24h for *Leptoria*, *Favites*; [86]), asynchronous spawning could occur at short temporal scale (*i*.*e*., several days) or between two consecutive moon cycles (28 days). Spawning events are determined by a combination of environmental conditions, including sea temperature or accumulated degree days, an empirical measure of heat requirements for growth and development, largely used in agronomy for crops or insect pests, or in aquaculture for egg incubation or gamete maturation (*e*.*g*., in mussels; [87]). In order to test whether distinct genetic clusters could result from asynchronicity related to different sea temperature regimes, we investigated the difference in cumulative degree days between the two remote sites (REU1 and REU4) from 6^th^ June 2015 to 18^th^ December 2015 (S4a Appendix; data provided by GIP RNMR/Marex). Over this time period, we estimated a mean difference of temperature of + 0.4°C per day between REU1 (warmer; mean temperature (± s.e.) = 25.0°C ± 0.087) and REU4 (cooler; mean temperature = 24.6°C ± 0.087). Over the 196 days of survey, the cumulative temperature difference between REU1 and REU4 reached 78.4°C. When extrapolating this difference over one year (taking the mean temperature for each site as a proxy for daily temperature for the 169 remaining days, *i*.*e*., a temperature difference of + 0.4°C per day between REU4 and REU1), we found a cumulative difference of 150.1°C between both sites (S4b Appendix). Assuming that an equal cumulative temperature threshold is needed for gametes to mature in both populations, this difference, for a given threshold, would correspond to a 6-days delay in gamete maturation between REU1 and REU4. Would this temperature difference, considering all the other interacting parameters, be sufficient to create a discrepancy in phenology and lead to a partial reproductive isolation between these two sympatric divergent lineages? To answer this question, more information on *P*. *damicornis β* reproduction in this part of the world (threshold temperature for gamete maturation, maturation start and duration, spawning event date) is obviously needed.

## Conclusions

While clonality has been documented for *P*. *damicornis β*, here we evidenced its clonal propagation for the first time in Reunion Island, South Western Indian Ocean. Indeed, around this island, asexual propagation plays a predominant role in populations of *P*. *damicornis β*, reproducing asexually by fragmentation and likely through the production of parthenogenetic larvae for long-distance dispersal (40 km). Thus, in case of high environmental and anthropogenic disturbances or global environmental changes, *P*. *damicornis β* populations from Reunion Island exhibiting low genotypic richness, such as those of La Saline-L’Ermitage lagoon, might be extremely vulnerable, implying dramatic consequences for the reef structure functioning.

## Supporting Information

S1 Appendix List of the loci used in this study. *Â*, the allelic richness, was estimated as the average number of alleles per locus on the basis of the smallest sample size (REU3, *N*_*MLG*_ = 13).
(PDF)

S2 Appendix Pareto distribution of *Pocillopora damicornis β* multi-locus genotypes for each site. (PDF)

S3 Appendix Plots of the mean Deviation Information Criterion (DIC) and the Δ(*K*) are presented both for the truncated dataset (a. and b., respectively) and for the entire dataset (c. and d., respectively). In each case, an arrow indicates the most likely number of clusters.
(PDF)

S4 Appendix (a) Water temperature in REU1 and REU4 lagoons from 6^th^ June 2015 to 18^th^ December 2015 and (b) cumulative day temperature for water lagoons in REU1 and REU4 over one year focusing on the last 40 days (from 7^th^ November 2015 to 18^th^ December 2015). (PDF)
