# Supplementary material for: Superclone Expansion, Long-Distance Clonal Dispersal and Local Genetic Structuring in the Coral Pocillopora damicornis Type β in Reunion Island, South Western Indian Ocean
Source: PLoS One. 2017 Jan 9;12(1):e0169692. doi: 10.1371/journal.pone.0169692 (PMC5222339; doi:10.1371/journal.pone.0169692)
Supplement: S1 Appendix — Â, the allelic richness, was estimated as the average number of alleles per locus on the basis of the smallest sample size (REU3, NMLG = 13). (PDF) [file pone.0169692.s001.pdf]

Table S1:

Panel	Locus Name	$\hat{A}$	Dye	References
1	Pd3-004	2.46	6-FAM	[41]
1	Pd3-005	4.65	NED	[41]
1	Poc40	6.07	6-FAM	[42]
1	PV2	3.02	VIC	[43]
1	PV7	2.52	VIC	[43]
2	Pd2-001	4.31	VIC	[41]
2	Pd2-006	3.10	NED	[41]
2	Pd3-008	3.65	6-FAM	[41]
2	Pd3-009	5.35	6-FAM	[41]
3	Pd3-EF65	4.21	PET	[28]
3	Pd4	2.54	6-FAM	[44]
3	Pd11	3.85	VIC	[44]
3	Pd13	4.21	NED	[44]
