# Supplementary figures and images for: Superclone Expansion, Long-Distance Clonal Dispersal and Local Genetic Structuring in the Coral Pocillopora damicornis Type β in Reunion Island, South Western Indian Ocean
Source: PLoS One. 2017 Jan 9;12(1):e0169692. doi: 10.1371/journal.pone.0169692 (PMC5222339; doi:10.1371/journal.pone.0169692)

REU1

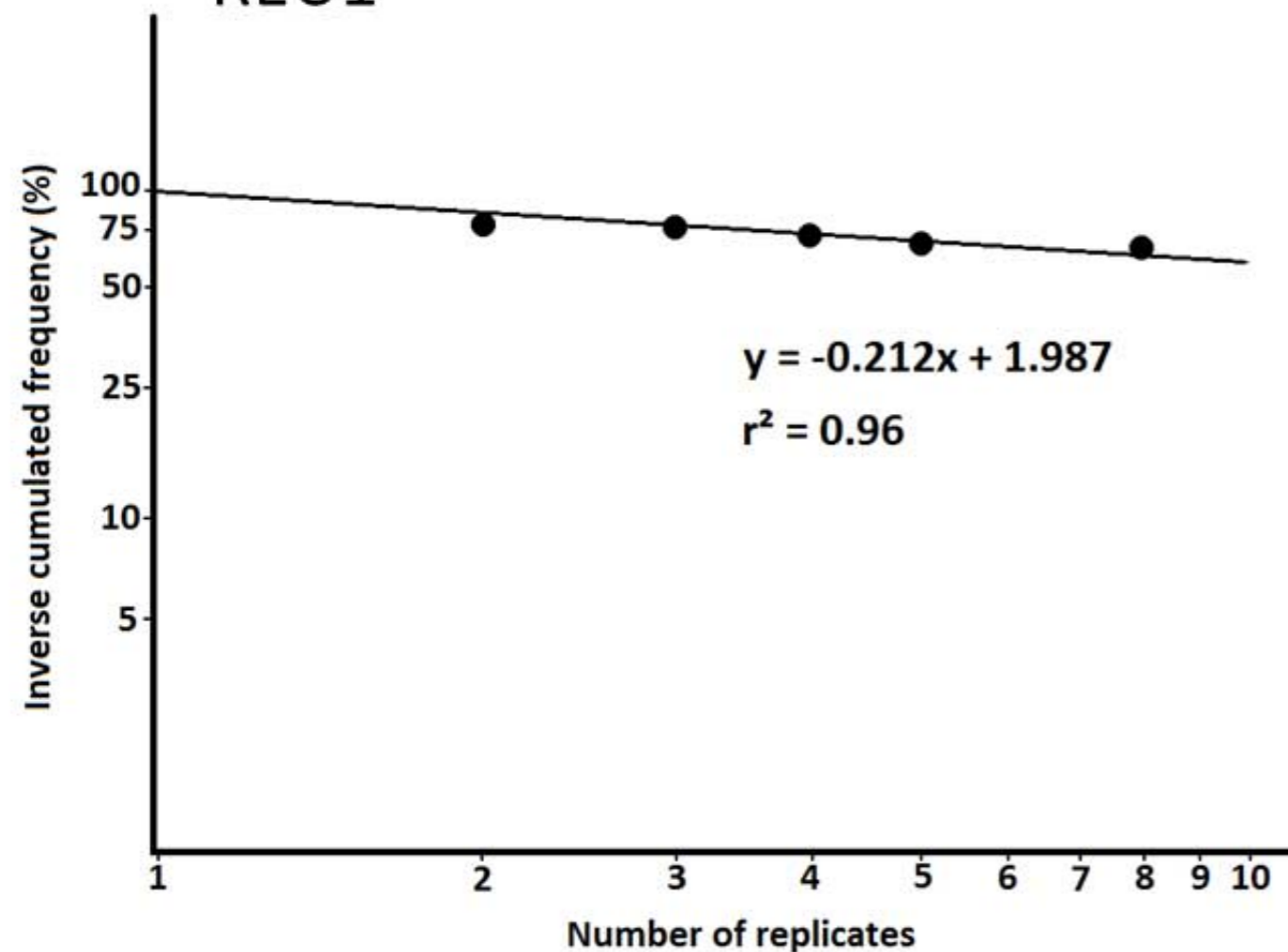

REU2

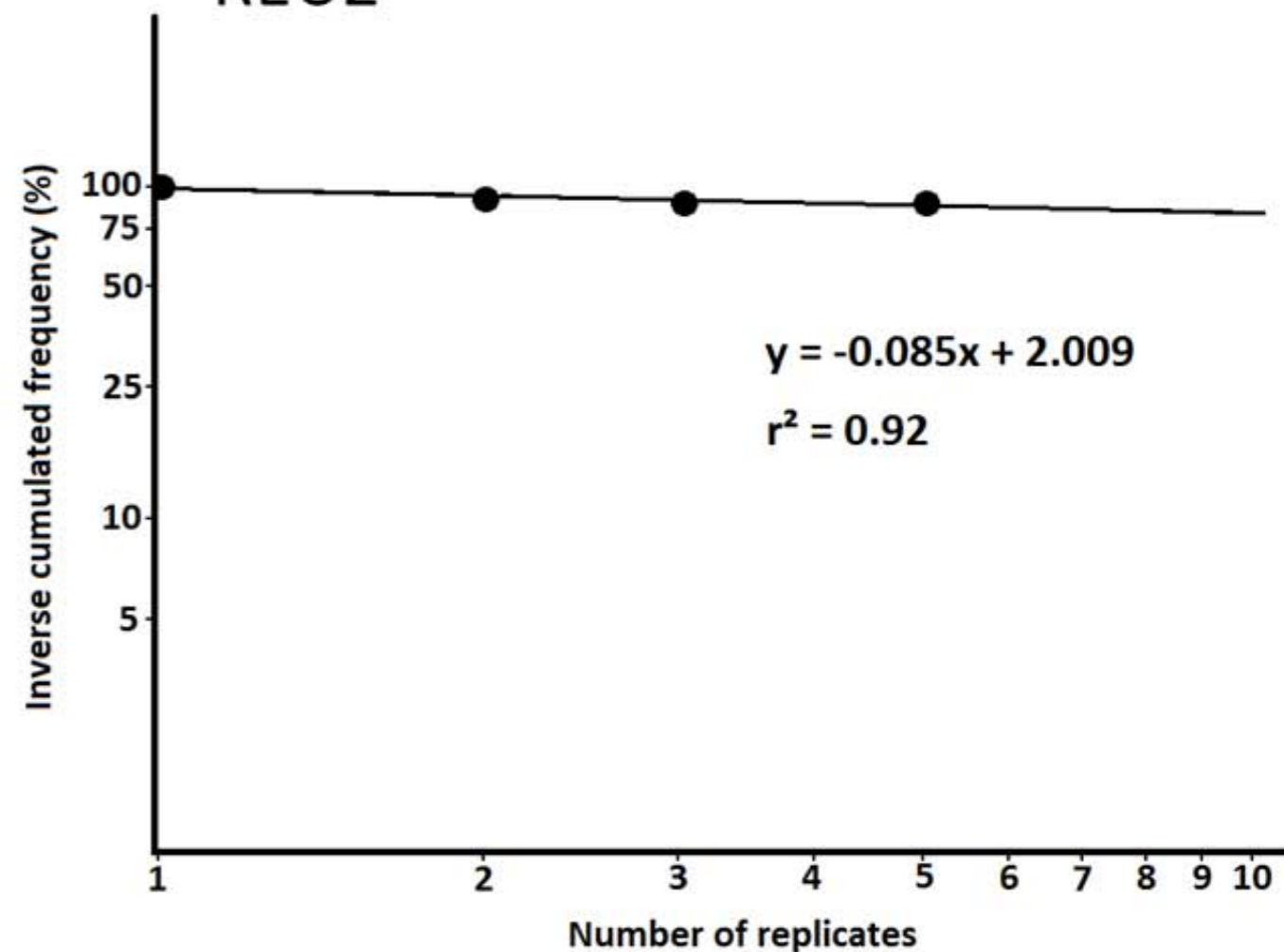

REU3

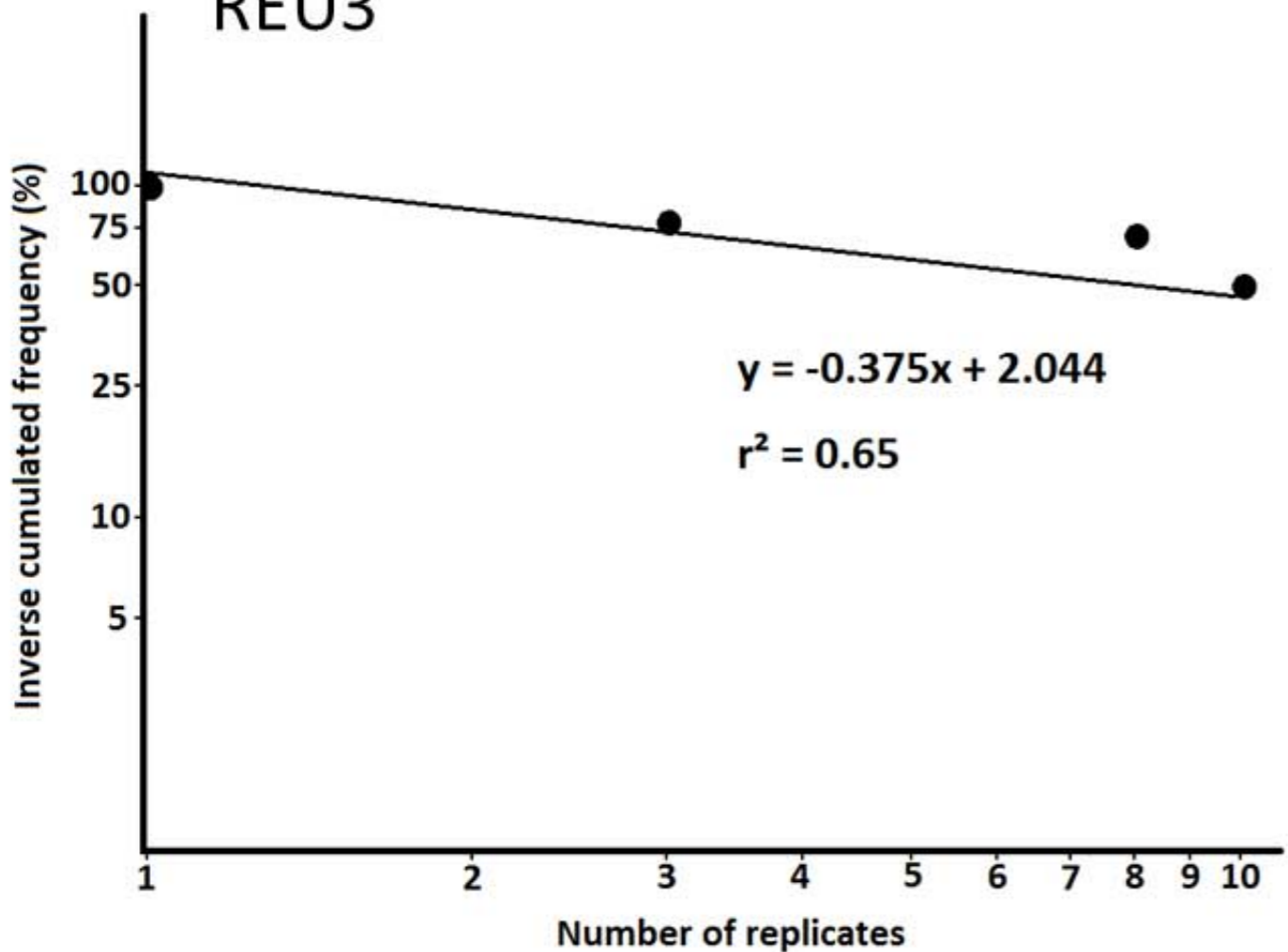

REU4

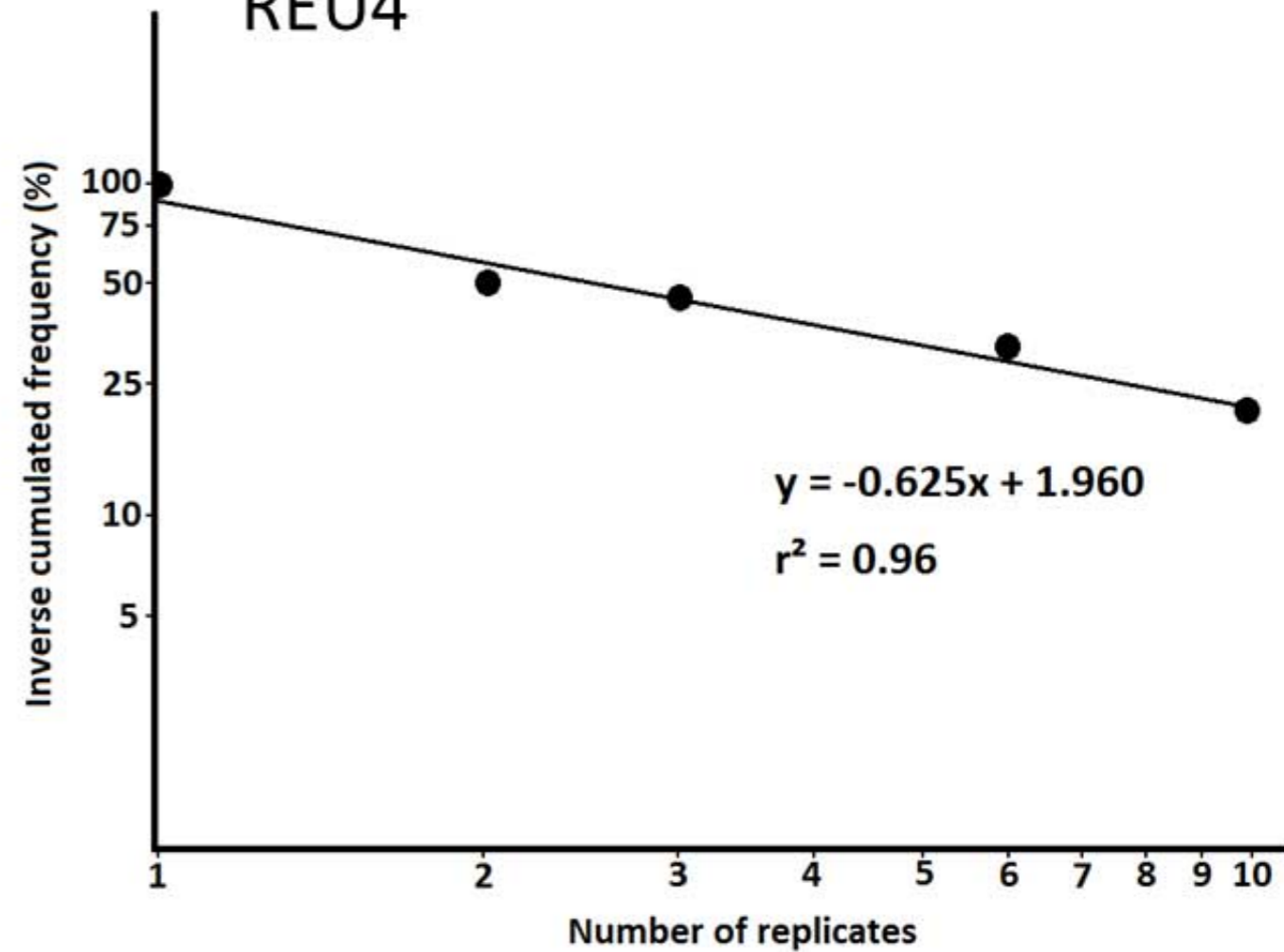

Supplement: S2 Appendix — (PDF) [file pone.0169692.s002.pdf]

Truncated  
dataset

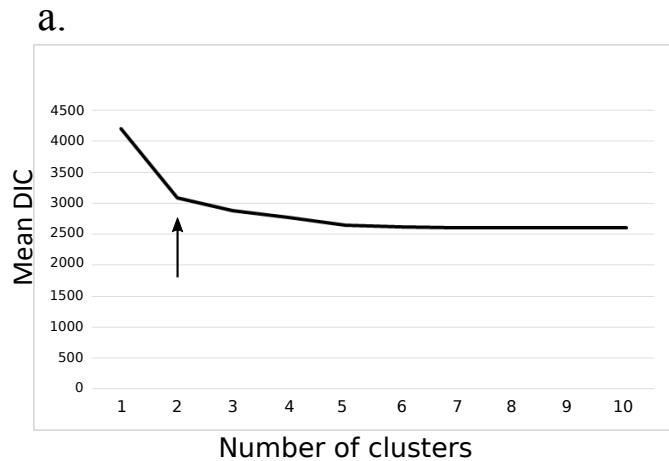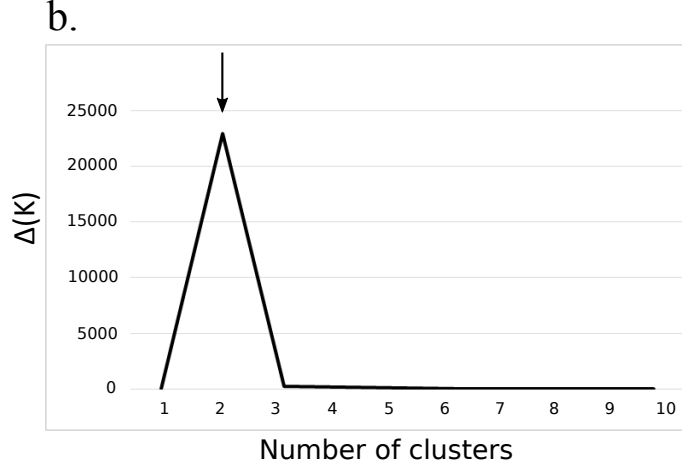

Entire  
dataset

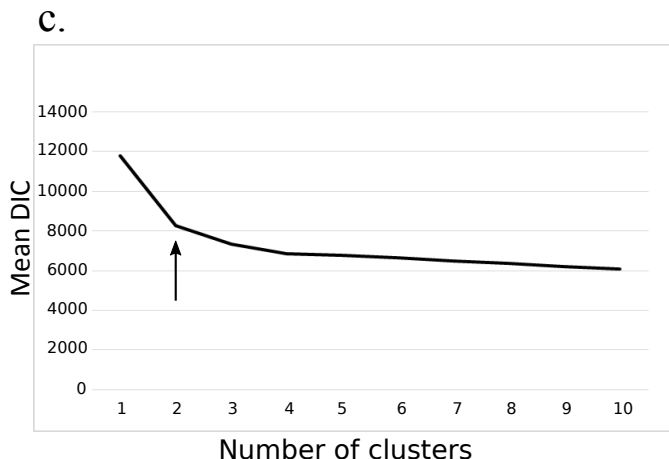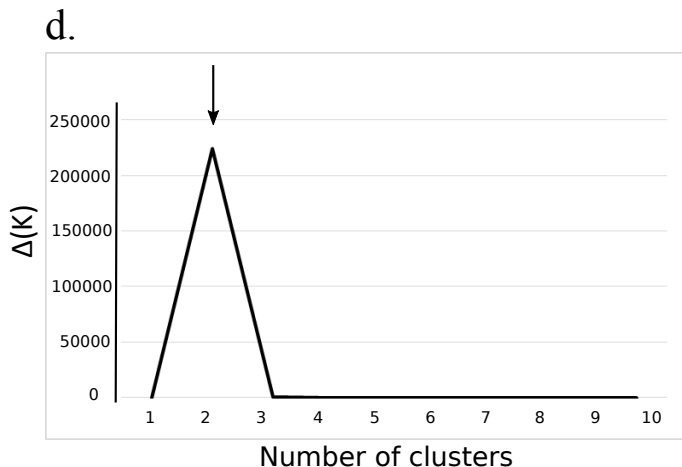

Supplement: S3 Appendix — In each case, an arrow indicates the most likely number of clusters. (PDF) [file pone.0169692.s003.pdf]

**a**

Water temperature (°C) in REU1 and REU4

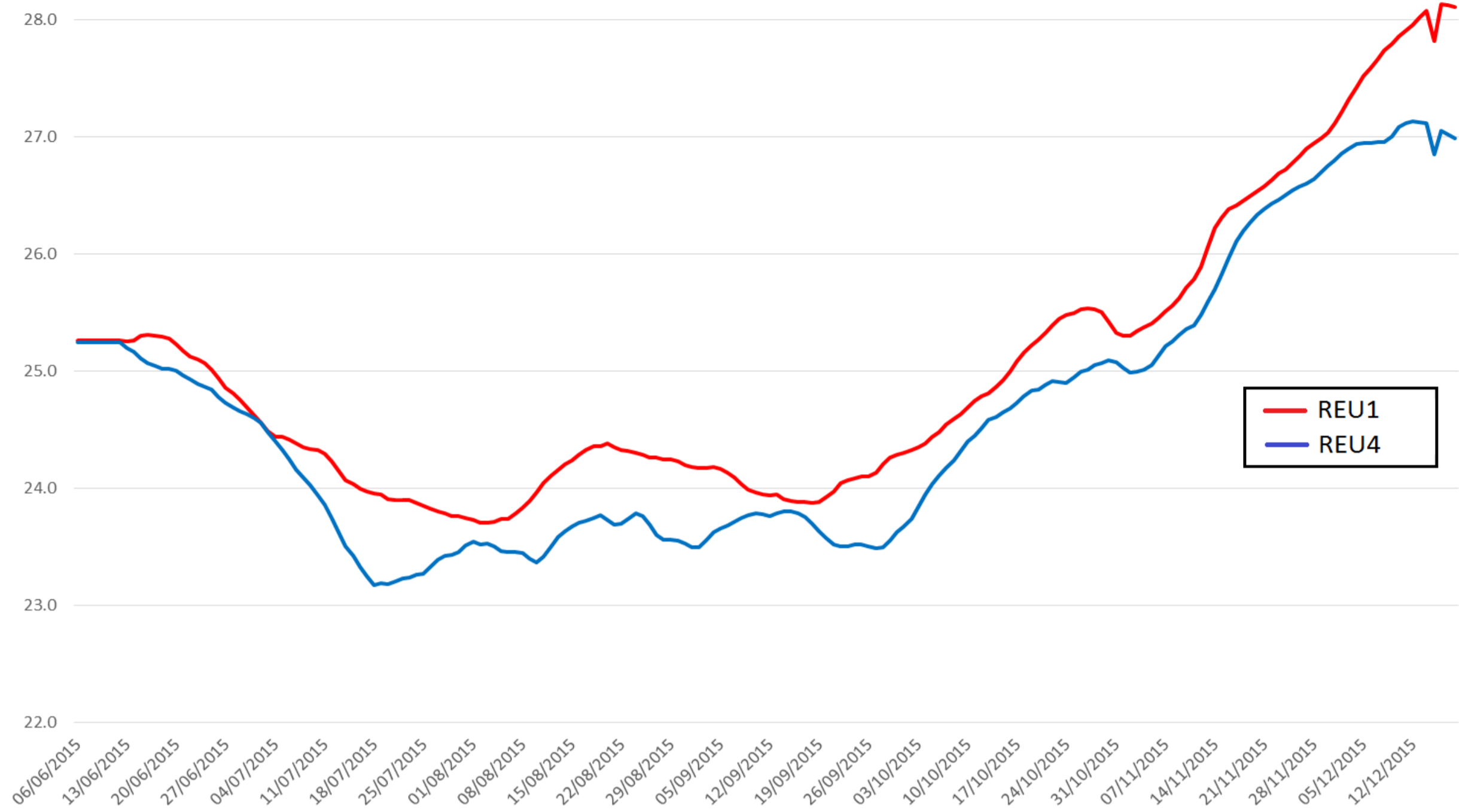**b**

Cumulative day temperature (°C) in REU1 and REU4

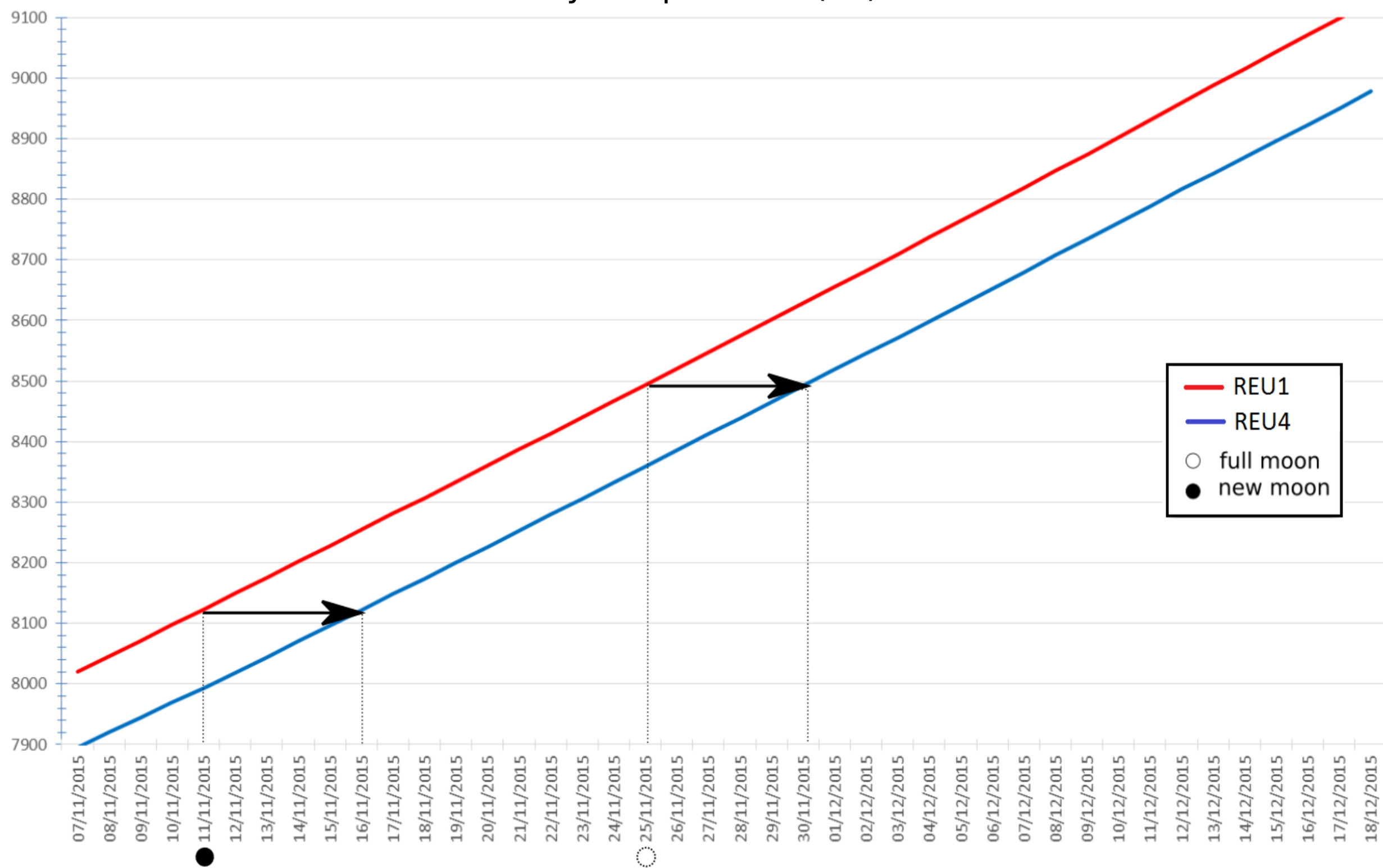

Supplement: S4 Appendix — (PDF) [file pone.0169692.s004.pdf]
